# Editorial: Biological soil crusts: spatio-temporal development and ecological functions of soil surface microbial communities across different scales

**DOI:** 10.3389/fmicb.2024.1447058

**Published:** 2024-07-30

**Authors:** Shubin Lan, David R. Elliott, Sonia Chamizo, Vincent J. M. N. L. Felde, Andrew D. Thomas

**Affiliations:** ^1^Key Laboratory of Vegetation Ecology of the Ministry of Education, Institute of Grassland Science, Northeast Normal University, Changchun, China; ^2^Jilin Songnen Grassland Ecosystem National Observation and Research Station, Northeast Normal University, Songyuan, China; ^3^Nature-Based Solutions Research Centre, University of Derby, Derby, United Kingdom; ^4^Desertification and Geoecology Department, Experimental Station of Arid Zones (EEZA, CSIC), Almería, Spain; ^5^Institute of Earth System Sciences, Section Soil Science, Leibniz University Hannover, Hanover, Germany; ^6^Department of Geography and Earth Sciences, Aberystwyth University, Aberystwyth, United Kingdom

**Keywords:** biocrusts, microbial community, ecological functions, habitats, recovery

Biological soil crusts (biocrusts) are widely distributed throughout the world and cover ~12% of the terrestrial surface (Rodriguez-Caballero et al., 2018). Composed of a diverse range of cyanobacteria, algae, lichens, mosses, and heterotrophic microorganisms (e.g., bacteria, fungi and archaea), biocrusts bind soil particles together to form a biological-soil matrix on the surface typically several millimeters thick (Green et al., 2012; Belnap et al., 2016; Zhang et al., 2018; Weber et al., 2022). Biocrusts are important sites of regional and global microbial diversity and perform multiple ecological functions (Lan et al., 2014; Belnap et al., 2016; Rossi et al., 2022; multifunctionality). Additionally, biocrust organisms were one of the earliest and most important groups involved in the evolution of terrestrial life and atmospheric chemistry before the widespread appearance of vascular vegetation (Lenton and Daines, 2017). Thus, they not only represent the early stages of terrestrial ecosystems, but also facilitate ecosystem development and succession (Beraldi-Campesi et al., 2014; Lan et al., 2022). Consequently, biocrusts are rightly seen as ecological engineers because of their role in the functioning and development of ecosystems (Viles, 2012) and because of their potential importance for the restoration of degraded terrestrial ecosystems (Bowker, 2007; Rossi et al., 2022).

Soil biocrusts are highly heterogeneous. This is reflected across both temporal and spatial scales, including at very small scales (Lan et al., 2019; Thomas et al., 2022). However, there are still large knowledge gaps regarding the composition of biocrust communities under different developmental states and habitat settings. We also have very little information on how organisms within biocrusts are spatially distributed or how they may interact with each other and with plants. The 24 articles that make up this Research Topic have been collated to promote our understanding of the heterogeneous development of biocrusts and their ecological multifunctionality in terrestrial ecosystems. Ultimately, our aim is to provide a scientific basis for the protection of the ecological functions of biocrusts at different scales, and to better inform the emerging field for using biocrusts in ecological restoration (e.g., cyanobacteria-induced biocrust technology).

## Biocrust community interactions and their ecological functions

Despite extensive studies documenting microbial communities found within biocrusts (e.g., Maier et al., 2018; Zhang et al., 2018), our understanding of their inter-relationships and ecological functionality remains limited. Addressing this knowledge gap in this Research Topic, Wang et al. and Elliott et al. demonstrate the distinctiveness and diversity of biocrust microbial groups at cm-scale depth resolution and reveal evidence of their co-existence and niche association. In particular, Elliott et al. integrated bacterial and fungal data of biocrusts from the Kalahari in Botswana and found that changes in the bacterial community were reflected by a corresponding change of fungal community, which is suggestive of probable cross-kingdom biotic interactions. Wang et al. further analyzed the relationship between bacteria, fungi, archaea and soil properties to provide unique insights into the specialist organisms of biocrusts and their niches. Various studies have employed sophisticated analytical techniques to probe the interactions of diverse microbes in biocrusts, yielding results that significantly advance our understanding of biocrust community assembly and their functional implications. For example, Liu et al., found that biotic interactions are strong drivers of community assembly, more so than environmental filtering, indicating that biocrust microbes play active roles in shaping their environments and are not merely followers of environmental selection pressures. This emerging knowledge poses significant challenges for integrating into land management strategies, particularly in the context of climate change and shifting land use patterns. It raises important questions about whether and how the microbial diversity within biocrusts should be preserved and managed to maintain ecosystem health and function.

In contrast to the studies exploring biocrust organisms and their interactions at the microscopic scale, other contributions to this Research Topic take a landscape-scale approach. For example, Li et al. present a comprehensive investigation of archaea across 3,500 km of northern China. Their research revealed that there are two dominant communities of archaea coexisting within biocrusts, characterized by species of *Haloarchaea* and *Nitrososphaeraceae*. These archaeal groups exhibit varied microbial interaction relationships, as evidenced by their assembly or co-occurrence patterns (e.g., assembled by drift or homogeneous selection). Together, these microbial communities are jointly regulated by taxonomic units, habitat types and geographical regions, although Liu et al. propose that deterministic processes of biotic interactions and environmental variables have greater effects on bacterial communities than fungal communities. Furthermore, Hansen et al. identified that overarching landscape features rather than vegetation and soil properties are the most crucial predictors shaping biocrust microbial communities. Evidently, these findings suggest that the activities of biocrust organisms at microscopic scales can have landscape scale outcomes, which are driven by complex and still poorly understood biotic interactions. Integrating our understanding of biocrust functions across these varying scales has been part of the aim for this Research Topic, and we are pleased to be able to present a broad range of research papers providing insights at different scales, and from different research perspectives.

Microbial communities and their biocrust structures have diverse ecological functions. One of the most important is to stabilize the soil by reducing wind and water erosion. This, in turn, will affect the geomorphological processes operating within landscapes, since stable land surfaces are a prerequisite for soil forming processes. For example, in a tropical degraded dry forest ecosystem, Szyja et al. found that biocrusts not only significantly reduced water infiltration, but also protected the most critical soil layers from water erosion. In rainfall simulation experiments on silty loam soils from a badlands area in Spain, Lázaro et al. demonstrated that the effectiveness of biocrusts in protecting soils from erosion varies with microbial and macroscopic community composition, which changes with biocrust development and/or succession. Additionally, Richardson et al., Hoellrich et al., and Tang et al. have shown that biocrust development and community type can also affect various biogeochemical cycles, such as carbon fixation/exchange, nitrogen fixation and NO_2_ release. Generally, more developed lichen- and/or moss-dominated biocrusts exhibit higher carbon and nitrogen fixation/exchange rates than those still at early stages (Housman et al., 2006; Maier et al., 2018). Thus, processes that lead to a resetting of biocrusts back to early developmental community types (for example, grazing pressures and disturbance) can significantly reduce carbon and nitrogen inputs to ecosystems. Furthermore, Baldauf et al. suggest that the ecological and hydrological functions associated with biocrust communities should be integrated into a spatially-explicit, process-based ecohydrological model. This integration is expected to be of great significance for accurately assessing and quantifying the observed ecological processes.

## Biocrust communities developed in diverse habitats

**Figure 1:**
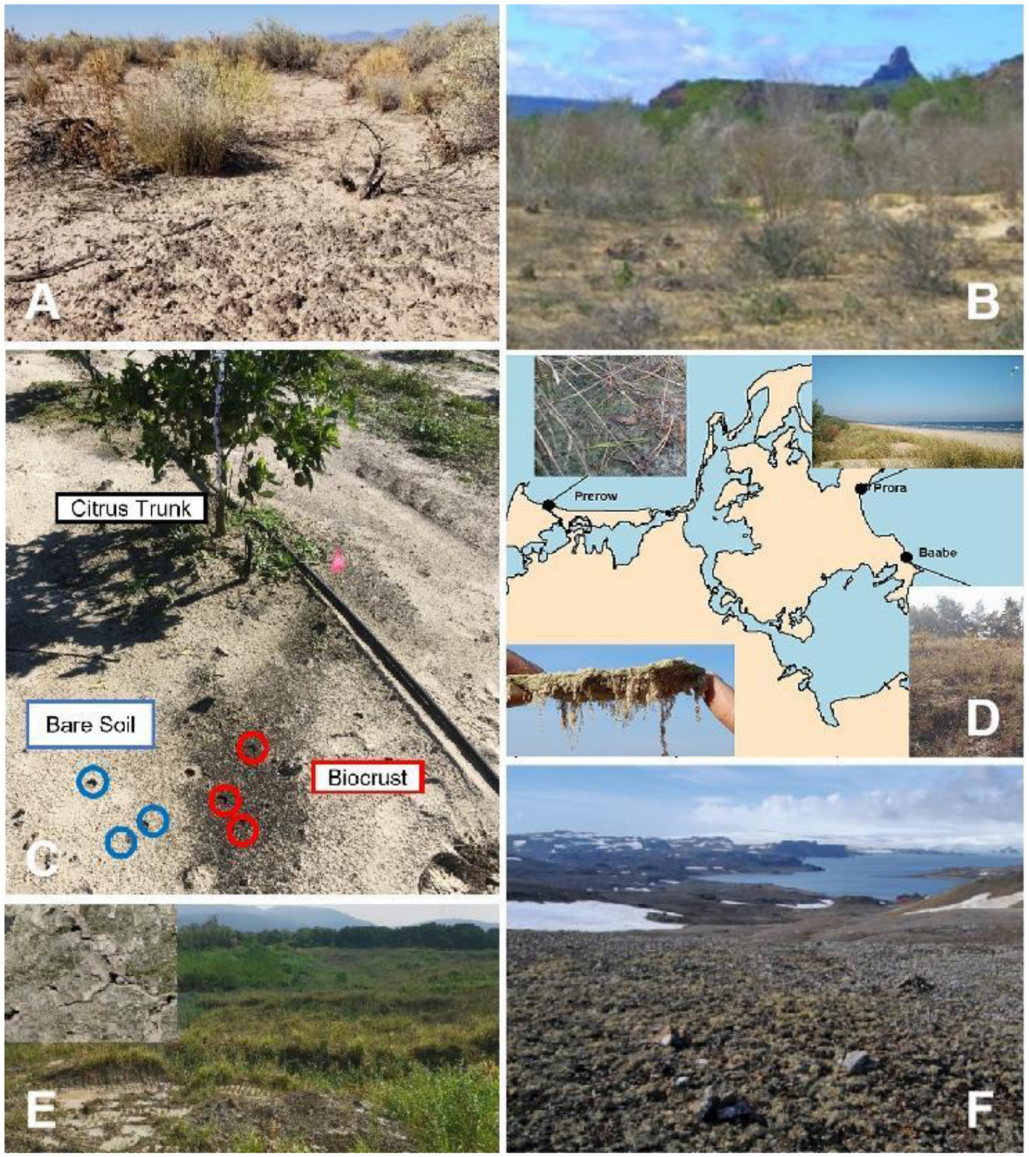
Biocrust communities in diverse habitat settings, including drylands [**(A)** Chihuahuan Desert; from Hoellrich et al.; **(B)** tropical dry forest of Caatinga; from Szyja et al.], agricultural land [**(C)** Florida citrus orchard; from Sorochkina et al.], coastal dunes [**(D)** along the Baltic Sea; from Glaser et al.], gold mine tailings [**(E)** Central China; from Xiao et al.], and Antarctica [**(F)** Maritime Antarctica; from Rybalka et al.].

Biocrust communities can be found on all continents, although most have been identified in dryland regions (e.g., Figures 1A, B; Belnap et al., 2016; Rodriguez-Caballero et al., 2018; Zhang et al., 2018). Acidobacteria, Actinobacteria, Bacteroidetes, Cyanobacteria and Proteobacteria are typically the major prokaryotic phyla (e.g., Hansen et al.; Tang et al.), whilst Ascomycota (e.g., Dothideomycetes) and Basidiomycota (e.g., Agaricomycetes) as the major eukaryotic phyla (e.g., Elliott et al.; Hansen et al.). Despite presumably periodic soil disturbance, Sorochkina et al. report nitrogen-fixing biocrust communities forming in a citrus orchard (Figure 1C). They quantified rates of fixation up to 3 mg N m^−2^ h^−1^, demonstrating their potential importance for the fertility of soils in these agro-ecosystems. Assuming 12.5 % of agricultural lands are covered by biocrust communities, they are estimated to supply 7–14 % of the total system nitrogen input, although this is still likely to be an underestimate (Sorochkina et al.).

As with many dryland dunes, coastal dune systems also are characterized by extreme conditions, such as intense solar radiation, substrate mobility, scarcity of nutrients, and strong winds. Additionally, coastal dunes are commonly associated with high salinity because of inputs from seawater and aerosol sprays. Although these conditions limit the growth and diversity of vascular plants, biocrusts are less constrained due to their unique physiological and ecological characteristics (e.g., Figure 1D; Glaser et al.). As far as we are aware, only a few studies have investigated biocrusts found on coastal dunes, describing their microbial biodiversity, hydrological properties and impacts on soil stabilization (Den Van Ancker et al., 1985; Kidron and Büdel, 2014; Schulz et al., 2016). Wang et al. report on the formation mechanism and community composition of biocrusts in the reef islands of the South China Sea. They show that biocrusts are dominated by cyanobacteria during the early stages of formation and provide evidence for the important role of cyanobacteria in the establishment and development of biocrusts on coastal dunes. There is also some evidence that biocrusts in coastal locations may recruit microbes from adjacent sandy soils, rather than supporting a general microbiome of biocrusts (Glaser et al.).

Arctic, Antarctic and alpine regions, characterized by their high latitude and/or altitude, can host extensive biocrusts in ice-free areas (at least temporarily) (e.g., Figure 1F; Pushkareva et al.; Rybalka et al.; Zhou et al.). In these environments, snowfall and ice often are typically the main water sources and are only available during periods warm enough for melting. Consequently, the active time of biocrusts in these settings is constrained by thawing intervals (Colesie et al., 2016; Williams et al., 2017; Weber et al., 2022). Upon hydration, Trexler et al. suggest that some of the biocrust community (e.g., Chitinophagaceae and some Firmicutes) rapidly activate within a few hours, while many others (e.g., Actinobacteria and Proteobacteria) remain inactive even 21 h after wetting. In these environments, Zhou et al. observed a prevalence of Bacteroides, a phylum known for its cold tolerance, although biocrusts developed in these areas are typically dominated by photoautotrophic cyanobacteria and microalgae. For example, Barrera et al. (2022) found that the most abundant cyanobacteria in Admiralty Bay, Antarctica were filamentous, with species from Nostocales, Oscillatoriales, and Pseudanabaenales dominating. In addition, Rybalka et al. found the microalgal communities in King George Island, Antarctica were dominated by Chlorophyceae, Trebouxiophyceae, Ulvophyceae, and Xanthophyceae. Not only are cyanobacteria and microalgae the main source of organic matter to the young soils of these areas (Mergelov et al., 2018), they also warm the surface because the dark pigmentation of microbial secretions absorbs more solar radiation (Couradeau et al., 2016), thus promoting soil development (e.g., Barrera et al., 2022; Rybalka et al.).

Xiao et al. and Schultz et al. have shown that biocrusts can also develop on mine tailings (Figure 1E), despite numerous physio-chemical challenges such as a poor soil structure, low nutrient status, and metal toxicity (Cabala et al., 2011). Although mining areas and tailings are distributed across climatic zones and on different soil types, Xiao et al. found the dominant microbes in biocrusts from these areas were similar to those in dryland biocrusts. This hints at the universal significance of dominant species (mainly cyanobacteria) in the formation of biocrusts, and also provides insights for their management and their restoration from disturbance. The collection of papers in this Research Topic, together with previously published studies (e.g., Williams et al., 2017; Weber et al., 2022), clearly demonstrate that biocrust communities can develop in diverse environments and colonize multiple habitats, and could therefore play an important role in ecological restoration.

## Biocrust community degradation and recovery

Although biocrusts develop in a variety of habitats and perform important ecological, hydrological and pedological functions with regional and even global significance, they are fragile and susceptible to changes in climate, land use and land management and disturbance (Housman et al., 2006; Rodriguez-Caballero et al., 2018). Consequently, a recent assessment by Rodriguez-Caballero et al. (2018) predicted that global biocrust coverage may be reduced by 25%−40% by the year 2070. Nevertheless, the sensitivity of biocrust organisms to environmental changes or human disturbances is not always the same, and there is a degree of decoupling between macroscopic lichens/mosses and microscopic microbial communities (e.g., Antoninka et al.; Palmer et al.). Macroscopic lichens/mosses on the surface may provide a buffer for microbial communities against environmental changes, and microbes that live in the subsoil, especially below one centimeter, are expected to be more similar regardless of the biocrust state and much less responsive to environmental changes (e.g., Palmer et al.). In addition, Hansen et al. showed that trampling was not a major driver of microbial community composition change in biocrusts of the Chihuahuan Desert, but rather the landscape features played a larger role in defining the biocrust community structure. This interesting finding offers an indication of the resilience/tolerance of some biocrusts to certain environmental change, which could be harnessed as part of sustainable land management planning and ecological restoration programs.

In some other scenarios, environmental changes and/or human activities can lead to biocrust disturbance and community degradation (Housman et al., 2006; Rodriguez-Caballero et al., 2018). Disturbances during dry seasons tend to be more destructive than those in rainy seasons, often reducing propagule quantity/availability, thus affecting the establishment and recovery of biocrusts (e.g., Jech et al.). Biocrust recovery begins with the establishment of biocrusts, which is influenced by a range of biotic and abiotic factors, and this process is generally limited by the colonizing ability of biocrust organisms such as cyanobacteria (Bowker, 2007; Ferrenberg et al., 2015; Faist et al., 2020). Natural recovery of biocrusts can start shortly after a disturbance, however, achieving full recovery to the original state is likely to take much longer, depending on the initial biocrust type/species composition (Belnap, 1993; Housman et al., 2006). Generally, biocrust communities dominated by cyanobacteria recover faster than those dominated by organisms such as lichens, which have slower growth rates (Green et al., 2012; Rubio and Lázaro). Nevertheless, when the extent/degree of disturbance is small, biocrusts can recover their stability within a short time. For example, Jech et al. observed that when the extent of disturbance on the Colorado Plateau was < 1 m^2^, the chlorophyll *a* and total exopolysaccharide content of biocrusts and soil stability fully recovered after 1.5 years. However, disturbance can have different degrees of severity and will consequently have different recovery times. Furthermore, based on a biocrust removal experiment in semiarid southeast Spain, Rubio and Lázaro showed that the recovery of biocrust organisms can be described by a sigmoidal function, where a relatively slow rate of recovery during the initial stages is followed by rapid growth, and ultimately a slow down as space or available resources become limited.

Unfortunately, relying solely on natural recovery in many settings is unlikely to fully restore soil biocrusts after disturbance. Therefore, researchers have explored various artificial methods to accelerate biocrust recovery, including physical soil stabilization, chemical addition and vascular plant establishment (Zhao and Wang, 2019; Chi et al., 2020; Adessi et al., 2021). However, concerns over the cost, sustainability and ecological safety of these measures mean that more direct restoration approaches such as inoculating degraded areas with biocrust organisms are often preferred (e.g., Tian et al.; Xiao et al.). In addition to cultivating biocrust organisms (e.g., cyanobacteria and/or mosses) artificially, there have been successes using well-developed biocrusts harvested from the field to seed degraded sites in order to promote soil surface stability and biocrust recovery (Bowker, 2007; Antoninka et al., 2020). Although this approach has proven effective in enhancing biocrust development in various studies (e.g., Schultz et al.), it requires the sacrifice of biocrusts in the donor site to benefit another and is costly. Nevertheless, this technology remains a practical option for achieving rapid biocrust recovery over relatively small areas, such as on contaminated mine tailings (e.g., Schultz et al.) or in areas undergoing specific human activities (e.g., quarries, road works and other construction sites).

In summary, the collection of articles in this Research Topic have clearly demonstrated the wide-ranging benefits of the heterogeneous and complex microbial communities contained within biocrusts. Specifically, they provide new insights into the interactions within biocrust communities across different scales, revealing that biocrusts are not passive elements within their environments but are active participants in shaping ecological processes in diverse habitats. These findings underscore the importance of biocrusts in global ecological processes and their potential in ecological restoration projects (e.g., natural recovery and artificial approaches to accelerate biocrust recovery). The insights gained from this Research Topic pave the way for innovative approaches to managing and restoring biocrusts, highlighting their significance in maintaining ecological balance and supporting global biodiversity.

## Author contributions

SL: Conceptualization, Formal analysis, Funding acquisition, Visualization, Writing – original draft, Writing – review & editing. DE: Formal analysis, Writing – original draft, Writing – review & editing. SC: Formal analysis, Writing – original draft, Writing – review & editing. VF: Formal analysis, Writing – original draft, Writing – review & editing. AT: Formal analysis, Writing – original draft, Writing – review & editing.
